# Dr. Christopher R. Finnegan

**Published:** 1913-05

**Authors:** 


Dr. Christopher R. Finnegan (college data not available) died in Buffalo, March 9, aged 28.

